# The safety and efficacy of the fissure-first approach in lung segmentectomy for patients with incomplete fissures

**DOI:** 10.3389/fonc.2024.1391835

**Published:** 2024-04-29

**Authors:** Shu-Sheng Zhu, Jianan Zheng, Liang Chen, Quan Zhu, Wei Wen, Jian Zhu, Jun Wang

**Affiliations:** ^1^ Department of Thoracic Surgery, Taizhou Hospital of Traditional Chinese Medicine, Taizhou, China; ^2^ Department of Thoracic Surgery, The First Affiliated Hospital with Nanjing Medical University, Nanjing, China; ^3^ Department of Thoracic Cardiovascular Surgery, General Hospital of Central Theater Command of the People’s Liberation Army, Wuhan, China

**Keywords:** lung segmentectomy, incomplete fissures, video-assisted thoracoscopic surgery, precision resection, minimally invasive surgery, fissure-first approach

## Abstract

**Background:**

Lung segmentectomy has gained much more attention as an important surgical method for treating early-stage lung cancer. However, incomplete fissures increase the difficulty of lung segmentectomy. The aim of this study was to analyze the safety and efficacy of the fissure-first approach in precision resection of lung segments for patients with incomplete fissures.

**Methods:**

The clinical data of patients with incomplete fissures who underwent lung segmentectomy were retrospectively analyzed. Date was divided into fissure-first approach in lung segmentectomy group (group A) and fissure-last approach in lung segmentectomy group (group B). The general linear data, operation times, intraoperative adverse events, postoperative recovery dates and complications were compared.

**Results:**

A total of 122 patients with complete clinical data were included. Patients in group B had more COPD (*p* < 0.05), and the lesions in group A were more closely related to the hilum of the lung (*p* < 0.05). Compared to Group B, Group A achieved better surgical outcomes, such as operation time, postoperative hospital stays, intraoperative bleeding, number of intrapulmonary lymph nodes sampled, counts of resected subsegments (except the upper lobe of the right lung), and rate of conversion to thoracotomy (all *p* < 0.05).

**Conclusion:**

The fissure-first approach is a safe and effective surgical approach in lung segmentectomy for patients with incomplete fissures. This approach can reduce the counts of resected subsegments and improve techniques in lung segmentectomy for patients with lung incomplete fissures.

## Introduction

In 1982, the American Lung Cancer Study Group (LCSG) designed the world’s first multicenter randomized controlled prospective study of sublobar resection and lobectomy for the treatment of lung cancer, LCSG821 (1). This study established lobectomy as the most important radical surgical procedure for treating lung cancer. With the popularization of lung computed tomography (CT), an increasing number of small pulmonary nodules have been screened and detected (2, 3). Moreover, with improvements in diagnosis and treatment technology (needle biopsy, surgery, fluid detection, etc.), additional small lung nodules have been subjected to pathological or genetic molecular diagnosis (4, 5). In addition, the TNM staging of lung cancer has been optimized in multiple ways (6, 7). So, interest in thoracic surgery for sublobar resection for early-stage lung cancer has increased again.

A comparative study of the effects of different surgical methods for early lung cancer, CALGB140503, JCOG0802 and JCOG0804, revealed that thoracoscopic segmentectomy could be performed for peripheral lung nodules and/or pure ground glass nodules ≤2 cm (8–10). It can achieve radical resection and is even better than radical lobectomy in terms of surgical effect-related indicators, such as the 5-year survival rate (8–10). Therefore, thoracoscopic lung segmentectomy has been rapidly included in surgical guidelines and expert consensuses for early-stage lung cancer in many countries (11, 12).

However, incidences of lung incomplete fissures (IFs) are important challenges frequently encountered by thoracic surgeons on lung segmentectomy. Data from previous studies indicate that IFs occurred in approximately 70% of right oblique fissures and 46% of left oblique fissures, while it occurs in 94% of right horizontal fissures (13, 14). Current evidence from the literature clearly shows that lung IFs increase the difficulty of lung surgery and increase the likelihood of prolonged air leakage and hospital stay during lung surgery (13, 14). In lobectomy, fissure-last approach was more recommended for patients with chronic obstructive pulmonary disease or complex cases with lesions near the hilum (15, 16). Segmental resection as a sublobar resection, the data is not clear. Therefore, the aim of this study was to compare and analyze the clinical efficacy of the fissure-first approach and fissure-last approach in lung segmentectomy for patients with lung IFs and to explore the optimal plan for this clinical challenge.

## Methods

### Study design and participants

A retrospective analysis was conducted on the clinical data of lung IFs patients who received lung segmentectomy in the department of thoracic surgery of the first affiliated hospital of Nanjing medical university (Jiangsu province hospital) from August 2020 to August 2023 by the same surgical team with rich experience on precision resection of lung segments and skilled operation experience. Lung segmentectomy was defined as (1) having a safe surgical margin distance which more than the maximum diameter of the tumor or 2 cm; (2) divided and severed the arterial, bronchial, and main venous structures (three are indispensable); (3) using the unit for lung subsegment, reduce the counts of resected subsegments as much as possible.

The 4-grade classification of lung IFs was used (17). The classification method was as follows. 1) Grade 0, with completely separated lobules and clear lung vascular structure; 2) Grade 1, completely obvious interlobar fissure at the visceral pleura, with more than 70% integrity of the interlobar fissure in the lung parenchyma; 3) Grade 2, some obvious visceral interlobar fissure; the integrity of the interlobar fissure in the lung parenchyma was 30%-70%; 4) Grade 3, no obvious interlobar fissure line, or the integrity of the interlobar fissure in the lung parenchyma was less than 30%. In clinical practice, incomplete fissures are easily dissociated by energy-based devices (ultrasonically activated scalpels and/or high-frequency electrocoagulable hooks) in patients with Grade 0 and Grade1 lung Ifs. And these procedures are simple and rarely leads to additional adverse results for surgical. Combined with the classification of previous literature (18), the date of patients with these two grades were excluded in this study.

Inclusion criteria: (1) the operation was performed by the surgeon with more than five years related experience in performing segmentectomy independently; (2) the number of segmentectomy operations performed by the surgeon in the past five years was more than 1000; (3) the operation was performed by lung segmentectomy; (5) the collected data should have intact surgical videos; (6) lung Ifs in patients must be at grade 3 or grade 4.

Exclusion criteria: (1) dealing with unplanned events for more than 30 minutes during the operation; (2) there are two or more lesions that need to be treated in the different anatomical fields; (3) cases with incomplete data, such as lack of key surgical details; (4) cases converted to lobectomy during the operation.

**Figure 1:**
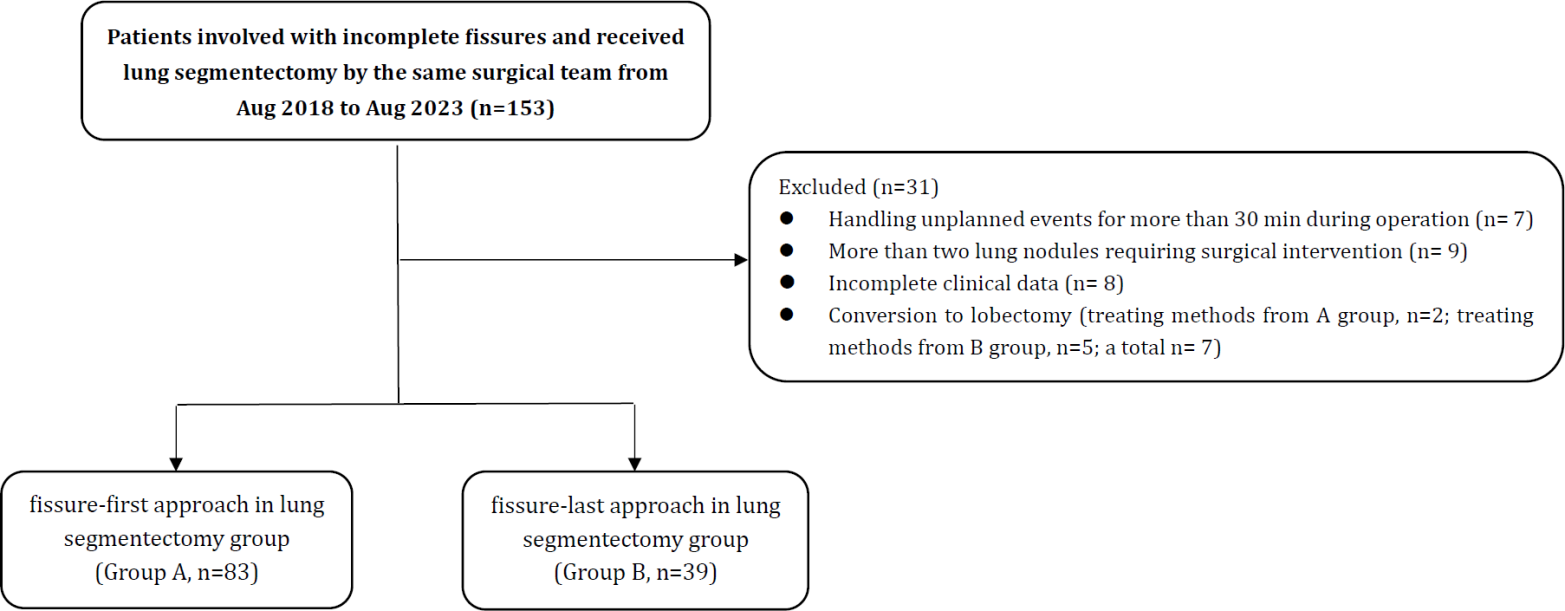
Flowchart of patients. There were 153 patients who met the inclusion and exclusion criteria, and a total of 122 patients were included in this study.

The included patients were divided into two groups according to the surgical approach using: fissure-first approach in lung segmentectomy group (group A) and fissure-last approach in lung segmentectomy group (group B). The specific case screening process is shown in **Figure 1**.

### Surgical procedures

**Figure 2:**
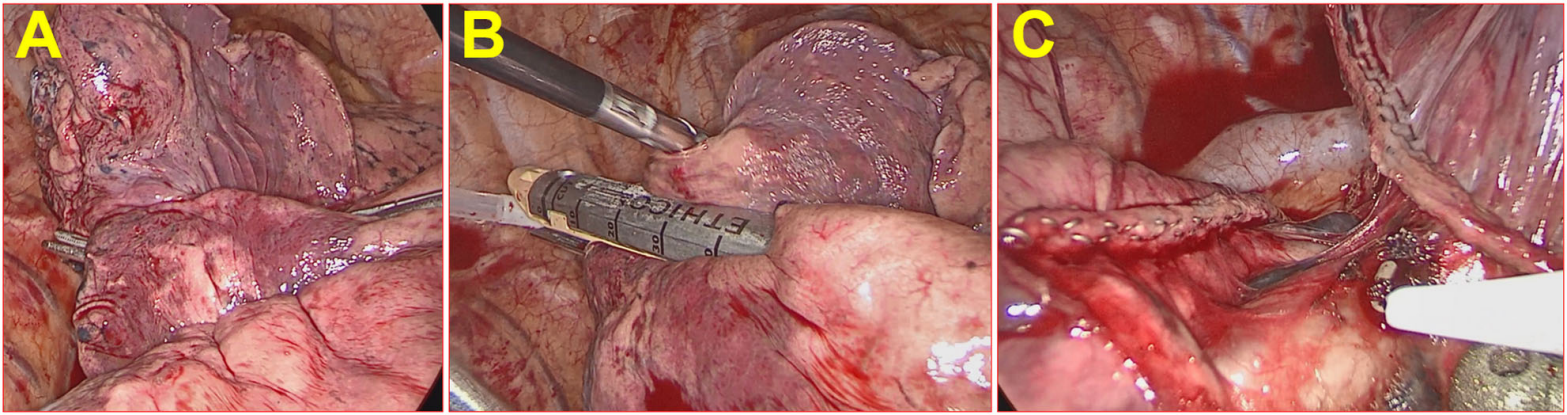
Fissure-first approach in lung segmentectomy on RS1+S2. **(A)** The right oblique fissure had some obvious visceral pleural interlobar fissures, and the integrity of the interlobar fissure in the lung parenchyma was approximately 40%. **(B)** Anatomical entry and exit were identified by vessels via the fissure-first approach. **(C)** A linear cutting stapler was used to cut/split and suture the fissure to achieve the fissure-first approach.

**Figure 3:**
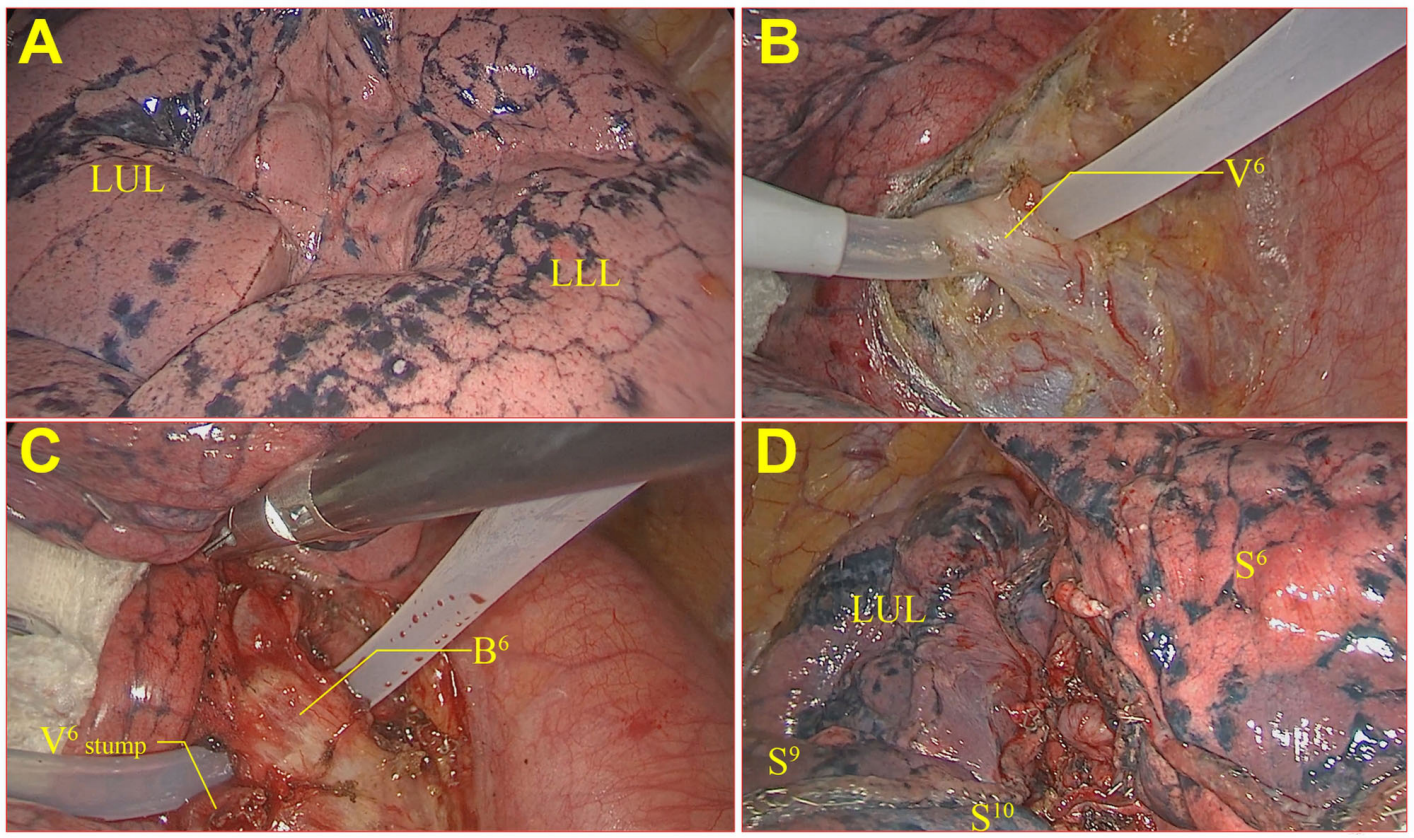
Fissure-last approach in lung segmentectomy on LS6. **(A)** The left oblique fissure was not an obvious interlobar fissure line; **(B)** V6 was dissociated, cut, and sutured; **(C)** B6 was dissociated, cut, and sutured; **(D)** The modified inflation−deflation method was applied for cone‐shaped cutting of the LS6.

Preoperative three-dimensional (3D) CT reconstruction planning, physical puncture localization of the nodules (positioning needle/methylene blue), and surgical incision design were carried out according to routine methods (19). The main surgical procedures of the fissure-first approach in the group A included opening the mediastinal pleura of the hilum of the lung and identifying the tunnel anatomical entry and exit by vessels for the fissure-first approach (such as A2b/A6a, A3a/A4 + 5, A4 + 5/A7 + 8 for the right lung, A1 + 2c/A6a, A1 + 2c/A6a, A4 + 5/A7 + 8 for the left lung) and segmental tracheas (anatomical location like segmental lung arteries). If necessary, the segmental veins were used to confirm this anatomy (such as V3b/V4 + 5 for the right lung). During these procedures, concomitant lymph node sampling was performed. When the above entrances and exits were opened with the assistance of energy-based devices to form the dissected hilar interlobar fissure line (tunnel), a linear cutting stapler was used to cut/split and suture the incision to achieve the fissure-first approach (see **Figure 2**). The surgical procedures about lung segmentectomy for the fissure-last approach for patients in the group B included the following steps (20, 21). Lung lesions in patients with lung Ifs on the lower lung were resected through the ligament approach to determine the segmental vein, segmental bronchus and segmental artery. And on the upper lobe lesions through dissecting the mediastinal pleura. Then dissociated the vein, such as RV3b, RV2, LV3, or LV4 + 5, which was lung vein approach. The exception is LS1 + 2, which is required to open the mediastinal pleura and dissect through the lung artery approach. The preoperative 3D CT reconstruction plan and the physical location of the nodule were compared, and the segmental artery, segmental bronchus, and segmental vein were segmented. The modified inflation−deflation method was routinely applied, and the opening the gate technique (22) was used for cone‐shaped targeted segments along the inboard inflation‐deflation demarcation of the lung segments (see **Figure 3**).

### Data collection and analysis

The dataset was partitioned into two distinct groups. The eligible medical records were subjected to comparative and analytical examinations encompassing various variables, including patient age, sex, nodule size (cm), nodule location, nodule solid component, nodule depth ratio, number of intrapulmonary lymph nodes sampled, surgical margin distance (cm), operation time (min), intraoperative bleeding (ml), number of conversions to thoracotomy, counts of lung subsegments resected, postoperative hospital stay (days), and occurrence of postoperative lung air leakage. The depth ratio method was used for the three symmetrical sectors (23).

The data are presented as the mean ± standard deviation for continuous variables and as absolute numbers and percentages for categorical variables. Categorical variables were assessed using χ2 tests or Fisher’s exact tests as appropriate, while *t* tests were conducted for continuous variables with independent samples. The nonnormally distributed data were compared between groups by *the* U test. A p value ≤0.05 indicated statistical significance. All the statistical analyses were performed using SPSS version 26.0 software.

## Results

### Patient characteristics

A total of 122 patients with complete data were included in this study. The patients had an average age of 56.59 ± 9.38 years and included 73 females (59.8%) and 37 smokers (30.3%). The mean diameter of the tumors was 1.13 ± 0.32 cm. A total of 83 patients were included in the group A, and 39 were included in the group B. In total, four lobes and three pathological types of lung nodules from patients who underwent segmentectomy were evaluated. According to the baseline data between the two groups, patients in group B had more chronic obstructive pulmonary disease (*p* < 0.05). The lesion location in group A was closer to the hilum of the lung (the depth ratio was greater than that in group B, *p* =0.0112), which indicated that the difficulty of precision resection of lung segments group increased in group A. There were no significant differences in the other baseline data, as shown in **Table 1**.

**Table 1:** The baseline characteristics data between the two groups of patients with incomplete lung fissures.

Variable	Total n=122	Group A n=83	Group B n=39	*p*
Age (year)	56.59 ± 9.38	55.96 ± 9.69	57.92 ± 8.65	0.2840
Sex (M/F)	49/73	34/49	15/24	0.7926
Smoking (no/yes)	85/37	58/25	27/12	0.9420
Comorbidity* (no/yes) Heart disease COPD Diabetes Hypertension Cerebrovascular	100/22 90/32 104/18 82/40 102/20	68/15 66/17 71/12 57/26 69/14	32/7 24/15 33/6 25/14 33/6	0.9868 0.0353 0.8929 0.6159 0.8365
Tumor size (cm)	1.13 ± 0.32	1.13 ± 0.30	1.12 ± 0.34	0.7383
Position RUL RLL LUL LLL	38 30 24 30	27 23 13 20	11 7 11 10	0.3428
CTR	0.41 ± 0.28	0.40 ± 0.29	0.41 ± 0.27	0.6423
Depth ratio	0.36 ± 0.11	0.38 ± 0.11	0.32 ± 0.07	0.0112

Group A, fissure-first approach in lung segmentectomy group; Group B, fissure-last approach in lung segmentectomy group; M, male; F, female; COPD, chronic obstructive pulmonary disease; RUL, right upper lung; RLL, right lower lung; LUL, left upper lung; LLL, left lower lung; CTR, consolidation tumor ratio.

### Surgical outcomes between the two groups

**Figure 4:**
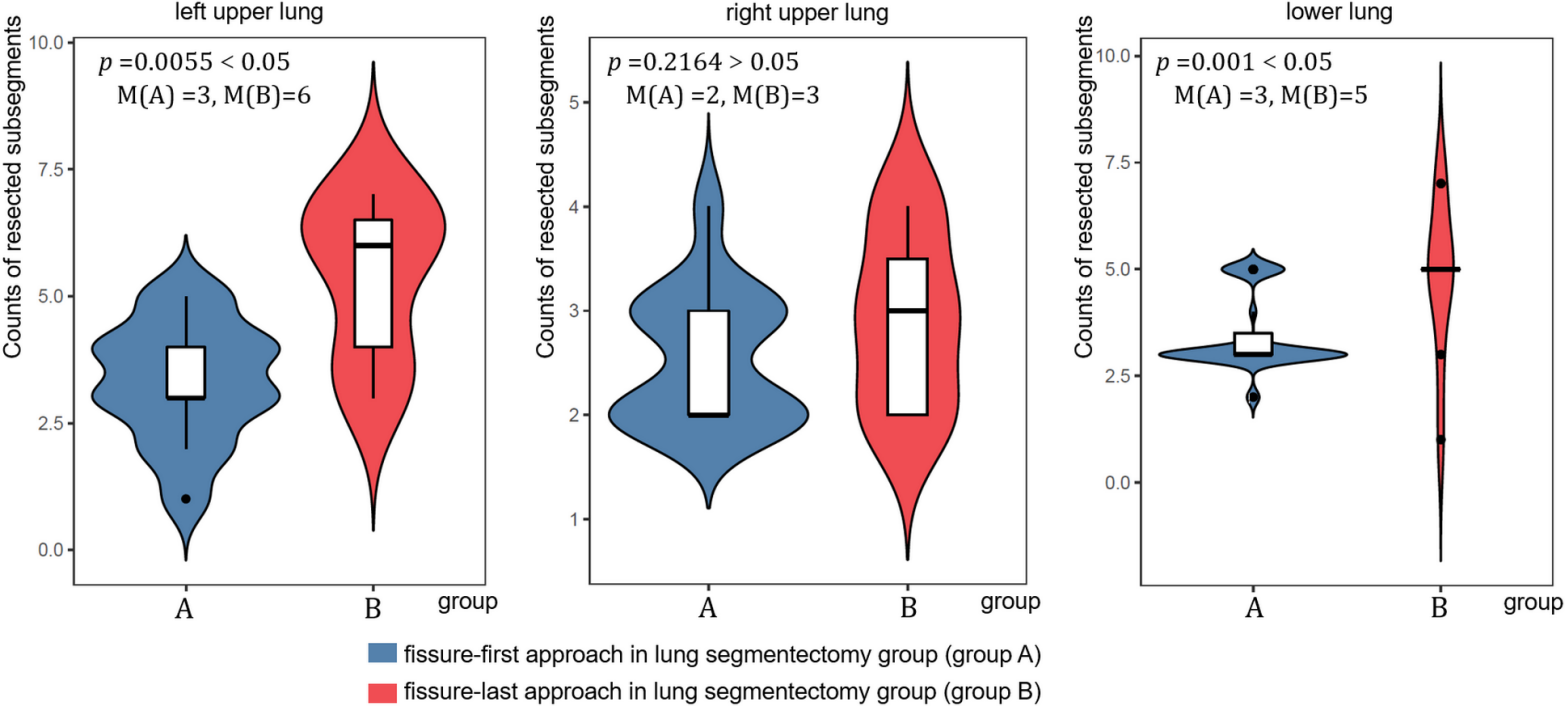
The counts of resected subsegments at different locations on the lungs in the two groups.

The surgical efficacy of lung segmentectomy was evaluated for patients in group A and group B for patients with lung IFs. The rate of conversion to lobectomy was lower in patients who planned to undergo the fissure-first approach than in patients who planned to undergo the fissure-last approach (χ^2 ^= 4.5869, *p* =0.0452). Among them, two patients were planned to undergo the fissure-first approach, and five patients were planned to undergo the fissure-last approach (see **Figure 1**); all seven of these patients had insufficient surgical margins. After excluding patients who were converted to lobectomy, the surgical efficacy data of group A were better than those of group B (operation time, surgical margin, rate of conversion to thoracotomy, intraoperative bleeding, postoperative hospital stay, and intrapulmonary lymph node sampling number; all *p* < 0.05). After excluding conversion to lobectomy, the postoperative air leakage rate was similar between the two groups (*p >*0.05) (**Table 2**). After excluding conversion to lobectomy, the counts of lung subsegments resected on the lower lung in group A was less than that in group B (M (group A) =3, M (group B) =5, *p* =0.001). Considering the special structure of the left upper lung, the upper lung was compared with the left and right lungs separately. The counts of lung subsegments resected on the left upper lung in group A was less than that in group B (M (group A) =3, M (group B) =6, *p* =0.0055), and counts of lung subsegments resected on the right upper lung in group A was similar to that in group B (*p* > 0.05) (**Figure 4**).

**Table 2:** Comparison of surgical outcomes between the two groups of patients with incomplete lung fissures.

Variable	Total n=122	Group A n=83	Group A n=39	*p*
Operative time (min)	121.68 ± 30.22	117.65 ± 29.10	130.26 ± 31.14	0.0407
Surgical margin (cm)	2.23 ± 0.56	2.30 ± 0.56	2.07 ± 0.55	0.0315
Pathology results AIS or others noncancerous nodule MIA IAC	36 62 24	23 42 18	13 20 6	0.3855
Conversion to thoracotomy (no/yes)	112/9	80/3	33/6	0.0293
Intraoperative bleeding (mL)	45.98 ± 23.38	42.65 ± 20.38	53.08 ± 27.71	0.0382
Postoperative hospital stays (day)	3.55 ± 0.83	3.41 ± 0.72	3.85 ± 0.99	0.0281
Air leakage (no/yes)	113/9	77/6	36/3	1.0000
Intrapulmonary LN sampling number	2.11 ± 0.76	2.27 ± 0.73	1.79 ± 0.73	0.0018
Positive LN number	0	0	0	–

Group A, fissure-first approach in lung segmentectomy group; Group B, fissure-last approach in lung segmentectomy group; AIS, adenocarcinoma in situ; MIA, minimally invasive adenocarcinoma; IAC, invasive adenocarcinoma cancer; LN, lymph node.

## Discussion

With the popularization of thin-slice CT scanning, the in-depth application of artificial intelligence and 3D reconstruction technology, an increasing number of small lung nodules have been detected in China (24–26). The main treatment for small lung nodules larger than 8 mm in diameter is surgical surgery, which mainly includes lobectomy, segmentectomy and wedge resection (27, 28). In recent years, segmentectomy has been considered to be a more advantageous surgical method for treating small lung nodules (6, 29, 30). However, not precision resection of lung segments often leads to inadequate surgical margins, especially in complex situations (31, 32). Moreover, studies comparing not precision resection of lung segments with lobectomy have yielded conflicting results (33, 34). Precision resection of lung segments should not include the volume of the lung segments only; after all, the volume at which the lung segmentectomy was performed can be replaced by that at large wedge resection (34, 35). Therefore, according to the anatomy of specific structures, a surgical method for precision resection of lung segments that meets the requirements of surgical margins with the lesion as the center is worthy of study.

In this study, we demonstrated the clinical efficacy of the fissure-first approach using lung segmentectomy for treating patients with IFs. Compared with those in the fissure-last approach group, the lesion location in the fissure-first approach group was closer to the hilum of the lung, which indicates that the difficulty of the operation was increased. Under more difficulty anatomical conditions, the fissure-first approach group has better surgical outcomes than the fissure-last approach, such as indicated by the operation time, surgical margin, rate of conversion to thoracotomy, intraoperative bleeding, length of postoperative hospital stay, and number of intrapulmonary lymph nodes sampled. In addition, when the lung lesion located in the lower lobe or the upper lobe of the left lung, the counts of lung subsegments resected can be reduced in patients who obtain a safe surgical margin via the fissure-first approach during lung segmentectomy. This reflects the connotation of lung segmentectomy. Notably, due to insufficient safe surgical margins, two patients were prepared for the fissure-first approach, which was excluded by conversion to lobectomy, in this study. And five patients occurred in the fissure-last approach. The conversion rate of the fissure-first approach was lower than that of the fissure-last approach (*p* < 0.05). Another result was also a premise of this study, there was no significant difference in the postoperative air leakage rate between the two groups (*p* > 0.05).

This retrospective analysis of the data showed that thoracic surgeons prefer the fissure-last approach when managing lung IFs patients with lung nodules who have chronic obstructive pulmonary disease. Because these patients often have emphysema, they are concerned that the fissure-first approach will increase the probability of air leakage after surgery by lobotomy thinking-inertia. However, the present study showed that the rate of postoperative lung air leakage was not greater in patients who received the fissure-first approach than in patients in the fissure-last approach group. This may be because lung segmentectomy is itself a sublobar resection, whereas lobectomy is an entire lobopleural structure. Therefore, we should not worry too much about air leakage after emphysema after lung segmentectomy via the fissure-first approach. Although, we admit it, more accurate clinical efficacy data need to be validated with big data.

This retrospective analysis of the data showed that patients with lung IFs who underwent lung segmentectomy using the fissure-first approach did not receive fewer count of resected subsegments in the right upper lobe. The researchers believe that the adjacent lobar fissures of the right upper lobe are S2 and S3, and the hilar structure of these two lung segments is easier to dissect.

In addition, this study emphasizes the superiority of the fissure-first approach. At present, anatomical sublobar resection has been gradually promoted for clinical application in thoracic surgery. The key technique of lung segmentectomy is to distinguish the anatomical structure of lung vessels and bronchi by 3D CT reconstruction technology (23, 36). Therefore, most thoracic surgeons who perform thoracoscopic lung segmentectomy begin with the fissure-first approach. To achieve precision resection of lung segments, especially in the early stages of the learning curve, one of the most practical techniques is to first dissect the interlobar fissure and then identify the vessels and bronchi.

This study has several limitations. This was a single-center retrospective study that included data from only patients who underwent surgery performed by the same surgical team to minimize confounding. Therefore, the results may be affected by selection bias.

## Conclusion

In conclusion, the fissure-first approach is a safe and effective surgical approach for patients with lung IFs who have undergone lung segmentectomy. This approach can reduce the count of resected subsegments and improve techniques for lung segmentectomy for patients with lung IFs. This technology provides a potential way to overcome the challenge of precise resection of complex anatomical lung segments. In addition, patients treated with the fissure-first approach recovered faster than patients treated with the fissure-last approach. Notably, the fissure-first approach in lung segmentectomy helps surgeons more clearly visualize the hilar structure, including the lung vessels and bronchi. Thereby, it improved the success rate of surgery for beginner thoracic surgeons with thoracoscopic lung segmentectomy.

## Data availability statement

The original contributions presented in the study are included in the article/supplementary material. Further inquiries can be directed to the corresponding authors.

## Ethics statement

The studies involving humans were approved by The Ethics Committee of the First Affiliated Hospital of Nanjing Medical University. The studies were conducted in accordance with the local legislation and institutional requirements. Written informed consent for participation was not required from the participants or the participants’ legal guardians/next of kin in accordance with the national legislation and institutional requirements.

## Author contributions

SZ: Conceptualization, Data curation, Funding acquisition, Writing – original draft. JNZ: Conceptualization, Methodology, Project administration, Visualization, Writing – original draft. LC: Project administration, Software, Supervision, Validation, Visualization, Writing – review & editing. QZ: Investigation, Methodology, Project administration, Resources, Writing – review & editing. WW: Conceptualization, Data curation, Funding acquisition, Methodology, Project administration, Writing – review & editing. JZ: Conceptualization, Formal analysis, Funding acquisition, Methodology, Software, Validation, Writing – original draft, Writing – review & editing. JW: Data curation, Funding acquisition, Methodology, Project administration, Resources, Software, Validation, Writing – review & editing.
